# Effect of hepatitis B virus DNA replication level and anti-HBV therapy on microvascular invasion of hepatocellular carcinoma

**DOI:** 10.1186/s13027-019-0219-8

**Published:** 2019-01-21

**Authors:** Chao Qu, Xinyu Huang, Kui Liu, Kun Li, Bin Tan, Linlin Qu, Jingyu Cao, Chengzhan Zhu

**Affiliations:** 1 Department of Hepatobiliary and Pancreatic Surgery, the Affiliated Hospital of Qingdao University, No.16 Jiangsu Road, Qingdao City, 266003 Shandong Province China; 2 Department of Infectious Diseases, the Affiliated Hospital of Qingdao University, No. 1677 Wutaishan Road, Qingdao City, 266555 Shandong Province China; 3 Medical College of Qingdao University, No.308 Ningxia Road, Qingdao City, 266071 Shandong Province China

**Keywords:** Hepatitis B virus DNA, Hepatocellular carcinoma, Microvascular invasion, Postoperative recurrence

## Abstract

**Background:**

Chronic hepatitis B virus (HBV) infection is a major risk factor for the occurrence and development of cirrhosis and hepatocellular carcinoma (HCC). Microvascular invasion (MVI) of HCC is closely related to postoperative recurrence. We aimed to investigate the effect of HBV DNA replication levels and anti-HBV treatment on the occurrence of MVI in HCC.

**Methods:**

A retrospective analysis of the clinical and pathological data of 660 patients undergoing hepatectomy for hepatocellular carcinoma at the Affiliated Hospital of Qingdao University from January 2015 to December 2017 is included in this study.

**Results:**

This study involved a total of 660 patients with an MVI incidence rate of 46.8% (309/660). Univariate analysis revealed that positive HBV surface antigen (HBsAg), detectable HBV DNA load, and administration of antiviral treatment were significantly associated with the formation of MVI. Multivariable logistic regression analysis in patients with positive seral HBsAg showed that detectable HBV DNA load (OR = 5.33, *P* < 0.001) was an independent risk factor for MVI. Antiviral treatment for more than six months (OR = 0.37, *P* = 0.002) was an independent protective factor against MVI. Patient groups with severe MVI had significantly higher rates of HBV infection (*P* = 0.017), a detectable HBV DNA load (> 100 IU/ml) rate (*P* < 0.001), and obvious low antiviral treatment rate (*P* = 0.021).

**Conclusions:**

HBV DNA replication level is an independent risk factors for the formation of HCC MVI, and anti-hepatitis B virus treatment has an inhibitory effect on MVI formation.

## Background

Hepatocellular carcinoma (HCC) is the fifth most common cancer and the third most common cause of cancer-related death in the world [1]. Although the purpose of radical treatment can be achieved by surgical removal of the tumor or liver transplantation, the high recurrence rate of HCC worsens the patient’s prognosis. HCC patients with tumor recurrence reached 70% or more after liver resection [2]. Among the various risk factors affecting the prognosis of liver cancer, microvascular invasiveness (MVI) has proved to be an independent predictor of high postoperative recurrence and low survival [3].

HBV-related HCC is one of the most common malignancies in the world, particularly in China [4]. Studies have shown that chronic hepatitis B virus (HBV) infection is a major risk factor for the development of cirrhosis and HCC [5]. High hepatitis B surface antigen (HBsAg) levels and high serum HBV DNA load were found to increase the risk of HBV-related cirrhosis and HCC significantly [6, 7]. Basic research found that HBV-initiated tumorigenesis may play a role in the development of HCC microvascular invasion [8, 9]. Recently, Lei Z et al. establish a nomogram for preoperative prediction of the presence of MVI in HBV-related HCC, which indicates that high HBV DNA load is independently associated with the development of MVI [10].

These findings suggest a potential correlation among hepatitis B virus infection, active replication, and development of microvascular invasion in HCC. However, whether antiviral treatment has an effect on the formation of MVI has not been reported in the literature. Therefore, we conducted this clinical study to explore the impact of hepatitis B virus DNA replication levels and anti–hepatitis B virus treatment on the formation of hepatocellular carcinoma microvascular invasion further.

## Methods

### Patients

This study included 660 HCC patients who underwent hepatectomy for hepatocellular carcinoma and pathological examination from January 2015 to December 2017 in the Affiliated Hospital of Qingdao University. The following patients were excluded: HCC associated with hepatitis C virus (HCV), preoperative history of arterial chemoembolization (TACE) or radiotherapy, non-radical resection or recurrence, and lack of complete clinical or pathological information. Finally, 660 patients were enrolled in the study, including 534 males and 126 females, with a male ratio of 80.9%. The average age was 56 years (range: 30–83 years). The following clinical data and pathology results from 660 patients are shown in Table 1.

**Table 1:** Univariate analysis of the impact of hepatitis B virus and tumor-related factors on the formation of MVI

Factors		MVI		P
		Yes	No	
Patient demographics				
Age	> 50 years	211	272	0.008
	<=50 years	98	79	
Gender	Male	262	272	0.017
	Female	47	79	
Antivirus treatment	Yes	67	105	0.016
	No	242	246	
Preoperative laboratory test				
HBsAg	Positive	278	297	0.041
	Negative	31	54	
HBsAb	Positive	31	23	0.104
	Negative	278	328	
HBeAg	Positive	77	86	0.901
	Negative	232	265	
HBeAg	Positive	126	142	0.933
	Negative	183	209	
HBcAb	Positive	300	337	0.527
	Negative	9	14	
Pre-S1Ag	Positive	234	268	0.851
	Negative	75	83	
HBV DNA load	> 100 IU/ml	166	145	0.001
	<=100 IU/ml	143	206	
AFP	> 7.2 ng/ml	259	203	0.001
	<=7.2 ng/ml	50	148	
CEA	> 3.4 ng/mL	71	81	0.976
	<=3.4 ng/mL	238	270	
CA125	> 35 U/mL	43	27	0.010
	<=35 U/mL	266	324	
CA19–9	> 39 U/mL	69	69	0.400
	<=39 U/mL	240	282	
CA24–2	> 15 U/mL	13	13	0.842
	<=15 U/mL	296	338	
ALB	> 35 g/L	258	311	0.057
	<= 35 g/L	51	40	
TBIL	> 22 umol/L	127	116	0.032
	<=22 umol/L	182	235	
ALT	> 50 U/mL	78	79	0.410
	<=50 U/mL	231	272	
AST	> 40 U/mL	111	75	0.001
	<=40 U/mL	198	276	
GGT	> 60 U/mL	133	97	0.001
	<=60 U/mL	176	254	
Pathological findings				
Edmonson grading	Grades III/IV	168	100	0.001
	Grades I/II	141	251	
Diameter	> 3 cm	224	196	0.001
	<=3 cm	85	155	
Number of lesions	Multiple	56	34	0.002
	Single	253	317	
Encapsulation	Incomplete	203	147	0.001
	Complete	106	204	
Presence of cirrhosis	Yes	219	213	0.006
	No	90	138	
Satellite nodules	Yes	73	21	0.001
	No	236	330	

The criterion for determining the positive values of the aforementioned data is based on the reference range of the normal values of the clinical data and the reference values reported in the relevant literature. Quantitative serum HBV-DNA replication levels were quantified by real-time quantitative polymerase chain reaction (PCR) with a detection limit of 100 IU/ml. Patients who received standard interferon therapy or who continued to use oral antiviral drugs for more than 6 months prior to surgery were classified into the antiviral therapy group (5 patients with interferons, 7 patients with lamivudine, and 30 patients with adefovir dipivoxil, 95 patients with entecavir, 21 patients with entecavir plus adefovir dipivoxil and 8 others).

This study was reviewed and approved by the Ethics Committee of the Affiliated Hospital of Qingdao University. All study participants, or their legal guardian, provided informed written consent prior to study enrollment.

### Diagnostic criteria of microvascular invasion

The definition of MVI is mainly based on the guideline of the HCC standardized pathological diagnosis published in 2015: no obvious intravascular thrombosis detected by imaging examination and macroscopic observation, and microscopic microvessels can be seen with tumor emboli or cancerous nests [11]. In this study, patients with MVI were divided into two groups according to the number and distribution of invasive vessels: patients with mild MVI (M1) had 1 to 5 affected blood vessels within 1 cm from the edge of the tumor; In the severe MVI group (M2), more than 5 vessels were involved in the 1 cm region from the edge of the tum or had invaded vessels located more than 1 cm from the tumor margin.

At the same time, each of the specimens was independently reviewed by two senior professional hepatobiliary pathologists to detect the presence and severity of MVI. If the diagnosis of the two pathologists is inconsistent, another pathologist with the same title will join the examination. If the examination result is consistent with one of the first two pathologists, the final diagnosis will be taken. When they are not consistent, the final diagnosis will be determined through the discussion of the pathology expert group.

### Statistical analysis

All calculations were performed by SPSS 19.0 software. Pearson chi-square test and Fisher’s exact test were used to compare the classified data. A multivariate binary logistic regression analysis was performed on variables of significance in the one-way ANOVA results to assess the relationship between the presence of microvascular invasion and variables. Using the stepwise regression method, the levels of the selected variable and the excluded variable were respectively, *P* = 0.05 and *P* = 0.10 to determine the independent risk factors and protective factors affecting MVI formation. Because viral factors (including HBV DNA load, the presence of cirrhosis, and the use of antiviral therapy) are only meaningful in HBV-infected patients, only HBsAg-positive patients were included in the multivariable analysis. The Kruskal-Wallis test was used to analyze the relationship between the severity of MVI and related factors of hepatitis B. The analysis of HBsAg factors included all patients, but other factors only included HBsAg-positive patients. Inspection level: α = 0.05.

## Results

### General data analysis

In this study, there were 272 males and 79 females in the MVI-negative group, with a male ratio of 77.5%, an average age of 57 years old (ranging from 31 to 83 years old); the MVI-positive group included 262 males and 47 females, with a male ratio of 84.8% and an average age of 55 years old (ranging from 30 to 82 years old). About 87.1% (575/660) of the patients had HBV-related HCC, and the remaining 85 patients (12.9%) were negative for HBsAg. A total of 311 patients (47.1%) had a detectable HBV DNA load (> 100 IU/ml), of which 253 (38.3%) had an HBV DNA load > 1000 IU/ml. A total of 168 patients (25.5%) was included in the antiviral therapy group. The incidence of microvascular invasion was 46.8% (309/660).

### Univariate analysis of the impact of hepatitis B virus and tumor-related factors on the formation of MVI

Univariate analysis showed that the presence of virus-associated serum markers, including HBsAg-positive (*P* = 0.04) results,detectable HBV DNA load (*P* < 0.001), and the presence of cirrhosis (*P* = 0.006), were significantly associated with the formation of MVI. In contrast, patients who received antiviral therapy (*P* = 0.016) had a significantly reduced risk of developing MVI (Table 1). Other tumor-related factors also showed significant differences between the two groups: (1) gender (*P* = 0.008), age (*P* = 0.017, 2) results of preoperative serological tests alpha-fetoprotein (AFP) (*P* < 0.001), CA125 (*P* = 0.010), total bilirubin (*P* = 0.032), glutamic acid transaminase (*P* < 0.001), γ-glutamyl transpeptidase (*P* < 0.001); and (3) Edmondson-Steiner histology grade (*P* < 0.001), tumor maximal diameter (*P* < 0.001), tumor number (*P* = 0.002), encapsulation (*P* < 0.001), existence of satellite nodules (*P* < 0.001).

### Multivariable binary logistic regression analysis of independent influencing factors of MVI in patients with positive seral HBsAg

The multivariable logistic regression method was adopted to analyze predictive factors of MVI in patients with positive seral HBsAg. As shown in Table 2, detectable serum HBV DNA load (OR = 5.33, *P* < 0.001) was an independent risk factor for microvascular invasion of hepatocellular carcinoma in multivariable regression analysis. Anti–hepatitis B virus treatment for more than six months (OR = 0.37, *P* = 0.002) was an independent protective factor in microvascular invasion of hepatocellular carcinoma. In addition, tumor-related factors, including male patients (OR = 2.16, *P* = 0.004), AFP levels > 7.02 ng/ml (OR = 2.86, *P* < 0.001), tumor size > 3 cm (OR = 2.20, *P* < 0.001), Edmondson-Steiner histological grade III/IV (OR = 2.97, P = *P* < 0.001), tumor encapsulation incompleteness (OR = 1.96, *P* = 0.001), and presence of satellite nodules (OR = 3.60, *P* < 0.001) were significantly and independently related to the formation of microvessel invasion of cell carcinoma.

**Table 2:** Multivariable logistic regression analysis of factors predictive of MVI in patients with positive seral HBsAg

Variables	OR	95% CI	*P* value
Gender (Male vs Female)	2.16	1.29–3.63	0.004
Antivirus treatment (Yes vs. No)	0.37	0.19–0.70	0.002
Seral HBV DNA load (> 100 IU/ml vs. < = 100 IU/ml)	5.33	2.88–9.86	0.001
AFP (> 7.02 ng/ml vs. <=7.02 ng/ml)	2.86	1.80–4.55	0.001
Edmonson Grade (Grades III/IV vs. Grades I/II)	2.97	1.99–4.43	0.001
Diameter (> 3 cm vs. <=3 cm)	2.20	1.45–3.33	0.001
Encapsulation (Incomplete vs. Complete)	1.96	1.32–2.93	0.001
Satellite nodules (Yes vs. No)	3.60	1.87–6.93	0.001

### Kruskal-Wallis test analysis of the relationship between the severity of MVI and factors associated with hepatitis B virus

Table 3 shows that the HBsAg-positive rate, detectable HBV DNA load (> 100 IU/ml), antiviral treatment rate, and presence or absence of cirrhosis were statistically significant among the MVI-negative group, M1 group, and M2 group. Among the severe-MVI groups, there was a higher HBsAg-positive rate (94.0%, *P* = 0.017) than in the mild MVI and MVI-negative groups, a higher detectable HBV DNA load (68.3%, *P* = 0.001), and a lower antiviral treatment rate (25.2%, *P* = 0.021). The proportion of patients with cirrhosis in the mild MVI group was the highest (80.0%).

**Table 3:** Relationship between MVI severity and HBV-related factors (The HBsAg factor included all 660 patients, and other factors included only HBsAg-positive patients)

HBV-related factors		Severity of MVI			P
		M0 (%)	M1 (%)	M2 (%)	
HBsAg	Positive	297 (84.6)	155 (88.6)	126 (94.0)	0.017
	Negative	54 (15.4)	20 (11.4)	8 (6.0)	
HBV DNA	> 100 IU/ml	145 (48.8)	82 (52.9)	84 (68.3)	0.001
	<=100 IU/ml	152 (51.2)	73 (47.1)	39 (31.7)	
Presence of cirrhosis	Yes	191 (64.3)	124 (0.80)	77 (62.6)	0.001
	No	106 (35.7)	31 (0.20)	46 (37.4)	
Antivirus treatment	Yes	102 (34.3)	41 (26.4)	31 (25.2)	0.021
	No	195 (65.7)	114 (73.6)	92 (74.8)	

## Discussion

The high recurrence rate of liver cancer is the result of a combination of factors. Many studies have shown that microvascular invasion (MVI) of liver cancer is an independent predictor of high postoperative recurrence and lower survival [3]. Some scholars have studied the prognosis of 249 patients undergoing liver tumor resection and found that microvascular invasion is one of the important indicators of recurrence of postoperative tumors [12]. In addition, some scholars who have studied surgical treatment of HCC patients have found that the microvascular invasion rate in patients with intrahepatic micrometastases reached to more than 30%, further demonstrating that liver cancer MVI are closely related to tumor recurrence after surgery [13].

Since early 1980s, several studies revealed that the relative risk of HCC is higher in HBsAg carriers compared to HBsAg seronegative populations [14, 15]. Molecular studies also showed the integration of HBV DNA into the host genome to cause genomic instability, which may lead to hepatocarcinogenesi [16, 17]. Among virus-derived quantitative markers, serum HBV DNA level was associated with increasing the risk of HCC in a dose-response relationship. The relative risk increased at the cut-off value of 2000 IU/mL [hazard ratio (HR): 2.3; *P* = 0.02] [18]. Therefore, there are critical relationships between the HBV status and the development/progression of hepatocellular carcinoma. In the Asia-Pacific region with the largest number of liver cancer patients, the hepatitis B virus (HBV) infection rate among HCC patients ranges from 70 to 90%, which is particularly serious in China [19]. A preoperative predictive model of MVI established by Lei et al. analyzed a large number of patients with HBV-related HCC (*n* = 1004) and found that high levels of HBV DNA load (> 10 * 4 IU/ml) were an independent predictor of the presence of MVI factor [10]. And Wei et al. also found that in patients with liver cancer, HBV infection is associated with the occurrence of vascular invasion [20]. These findings suggest a possible correlation between hepatitis B virus infection and active replication and the development of microvascular invasion in HCC. Our studies further confirmed the existence of this correlation and found inhibition of HCC microvascular invasion by anti-HBV treatment.

HBV-related factors (including high hepatitis activity scores and high HBV DNA load) were significantly associated with late recurrence, whereas antiretroviral drugs continued to lower the rate of late recurrence of HBV replication [21–23]. A randomized controlled trial by Lin et al. showed that patients who received antiviral therapy had significantly longer overall survival (93.8% vs. 62.2%) and relapse-free survival (55.6% vs 19.5%) [24]. This is consistent with our findings. Our results suggest that active HBV replication is associated with a higher rate of microvascular invasion in patients with HCC, and that patients with antiviral therapy for more than half a year have a lower likelihood of microvascular invasion, which is a good explanation for the anti-tumor effects of antiviral therapy. We can conclude that antiviral therapy inhibits HBV replication to reduce the incidence of MVI, which can reduce the invasiveness and metastatic potential of HCC, thereby reducing the risk of postoperative recurrence.

According to the current guidelines for anti-HBV recommendations based on HBV DNA replication levels, HBeAg-positive patients, with HBV DNA ≥20, 000 IU/ml, are recommended for antiviral therapy [25]. However, based on our findings in this study, the incidence of microvascular infiltration in HCC patients with undetectable HBV DNA load (≤100 IU/ml) was significantly lower than that in patients with detectable HBV DNA load (> 100 IU/ml). Therefore, for patients who meet the indications for antiviral therapy, antiviral therapy should be standardized and timely to reduce the replication levels of HBV DNA in order to reduce the incidence of MVI. At the same time, the guidelines recommend that patients with an objective basis for liver cirrhosis be identified. Regardless of alanine aminotransferase (ALT) and HBeAg conditions, active antiviral therapy is recommended. In Table 3, the liver cirrhosis rate was the highest in the M1 group, but the antiviral treatment rate was higher than that in the M2 group. Perhaps due to the presence of liver cirrhosis, more patients were able to receive timely antiviral treatment, thus lowering the MVI severity in formation. This also illustrates the effect of antiviral therapy on the severity of MVI from another perspective.

This study may not be able to reveal in detail how HBV infection and active replication contributes to the development of HCC microvascular invasion and how anti-hepatitis B virus treatment inhibits the formation of MVI. In addition, the differences in antiviral treatment regimens and the different surgical approaches patients used may have a slight impact on our results. However, despite these deficiencies, HBV infection and active replication were confirmed as independent risk factors for MVI formation. The protective effect of anti-HBV treatment on MVI formation was first discovered and analyzed in detail. Finally, this study only studied HBV-related HCC, and the conclusion does not apply to HCC caused by other liver viruses.

## Conclusions

In addition to the factors of the tumor itself, the HBV DNA replication level is an independent risk factor for the formation of MVI of HCC, and anti-hepatitis B virus treatment has an inhibitory effect on MVI formation. For HBV-associated HCC patients with a positive HBV DNA test, an increased risk of microvascular invasion should be recognized. Standardized, timely and effective anti-hepatitis B virus treatment may provide patients with a more promising prognosis.

## Abbreviations

ALT: Alanine aminotransferase
HBsAg: Hepatitis B surface antigen
HBV: Hepatitis B virus
HCC: Hepatocellular carcinoma
HCV: Hepatitis C virus
HR: Hazard ratio
MVI: Microvascular invasion
PCR: Polymerase chain reaction
TACE: Arterial chemoembolization
